# 

**Published:** 1892-08

**Authors:** 


### CONTRIBUTORS TO VOLUME XXXII.

Bartlett, Frederick W., M. D. . . . . . . . . . . . . . . . . . . . Buffalo.
Benedict, A. L., M. D. . . . . . . . . . . . . . . . . . . . . . . . Buffalo.
Cary, Charles, M. D. . . . . . . . . . . . . . . . . . . . . Buffalo.
Clark, Horace, M. D. . . . . . . . . . . . . . . . . . . . . . . . . Buffalo.
Crockett, M. A., M. D. . . . . . . . . . . . . . . . . . . . . . . . Buffalo.
Cushing, Clinton, M. D. . . . . . . . . . . . . . . . . San Francisco.
Daggett, B. H., M. D. . . . . . . . . . . . . . . . . . . . . . . . . Buffalo.
Dagenais, Alphonse, M. D. . . . . . . . . . . . . . . . . . . . . Buffalo.
Dowd, J. Henry, M. D. . . . . . . . . . . . . . . . . . . . . . . . Buffalo.
Dunham, Sydney A., M. D. . . . . . . . . . . . . . . . . . . . . . Buffalo.
Frederick, C. C., M. D. . . . . . . . . . . . . . . . . . . . . . . Buffalo.
Hayd, Herman E., M. D. . . . . . . . . . . . . . . . . . . . . . . Buffalo.
Himmelsbach, G. A., M. D. . . . . . . . . . . . . . . . . . . . . Buffalo.
Hubbell, Alvin A., M. D. . . . . . . . . . . . . . . . . . . . . . Buffalo.
Jones, Allen A., M. D . . . . . . . . . . . . . . . . . . . . . . . . Buffalo.
Jones, Mary A. Dixon, M. D. . . . . . . . . . . . . . . . . . . Brooklyn.
Krauss, William C., M. D. . . . . . . . . . . . . . . . . . . . . . Buffalo.
Lothrop, Thomas, M. D. . . . . . . . . . . . . . . . . . . . . . . Buffalo.
McMurtry, L. S., M. D. . . . . . . . . . . . . . . . . . . . . . Louisville.
Marcy, William L., Esq. . . . . . . . . . . . . . . . . . . . . . . Buffalo.
Manley, Thomas H., M. D. . . . . . . . . . . . . . . . . . . . New York.
Mann, Matthew D., M. D. . . . . . . . . . . . . . . . . . . . . . . Buffalo.
Mays, Thomas J., M. D. . . . . . . . . . . . . . . . . . . . . Philadelphia.
McAdam, L. J., M. D. . . . . . . . . . . . . . . . . . . . . . . . . Buffalo.
Mercer, A. Clifford, M. D. . . . . . . . . . . . . . . . . Syracuse.
Miller, John A., Ph. D. . . . . . . . . . . . . . . . . . . . . . . . Buffalo.
Milliken, Samuel E., M. D. . . . . . . . . . . . . . . . . . . New York.
Mynter, Herman, M. D. . . . . . . . . . . . . . . . . . . . . . . . Buffalo.
Park, Roswell, M. D. . . . . . . . . . . . . . . . . . . . . . . . . Buffalo.
Parmenter, John, M. D. . . . . . . . . . . . . . . . . . . . . . . . Buffalo.
Peterson, Frederick, M. D. . . . . . . . . . . . . . . . . . . . New York.
Potter, William Warren, M. D. . . . . . . . . . . . . . . . . . . Buffalo.
Price, Joseph, M. D . . . . . . . . . . . . . . . . . . . . . . . Philadelphia.
Pryor, John H., M. D. . . . . . . . . . . . . . . . . . . . . . . . . Buffalo.
Putnam, James W., M. D. . . . . . . . . . . . . . . . . . . . . . . Buffalo.
Putnam, Hon. James O. . . . . . . . . . . . . . . . . . . . . . . . Buffalo.
Reed, Charles A. L., M. D. . . . . . . . . . . . . . . . . . . . Cincinnati.
Richmond, Charles H., M. D. . . . . . . . . . . . . . . . . Livonia, N. Y.
Richmond, Nelson G., M. D. . . . . . . . . . . . . . . . . Fredonia, N. Y.
Riley, Henry A., A. B., LL. D. . . . . . . . . . . . . . . . . . New York.
Ross, James F. W., M. D . . . . . . . . . . . . . . . . . . . . . . Toronto.
Smith, Eugene A., M. D. . . . . . . . . . . . . . . . . . . . . . . Buffalo.
Stockton, Charles G., M. D. . . . . . . . . . . . . . . . . . . . Buffalo.
Storrs, M., M. D. . . . . . . . . . . . . . . . . . . . . . . . . . Hartford.
Thornbury, Frank J., M. D. . . . . . . . . . . . . . . . . . . . . Buffalo.
Weigel, Louis A., M. D. . . . . . . . . . . . . . . . . . . . . Rochester.
Wende, Ernest, M. D. . . . . . . . . . . . . . . . . . . . . . . . . Buffalo.
Williams, Herbert U., M. D. . . . . . . . . . . . . . . . . . . . . Buffalo.
American Surgical Association.
Buffalo Academy of Medicine.
Medical Society of the County of Erie.
Medical Society of the State of New York.
Mississippi Valley Medical Association.
Pan-American Medical Congress.
Philadelphia County Medical Society.

### LIST OF ILLUSTRATIONS.

ON THE DIGESTIVE FERMENT OF CARICA PAPAYA IN GASTRO-INTESTINAL DISORDER. (FRANK WOODBURY, M. D.)

Specimens of Papoid Artificial Digestion . . . . . . . . . . . . 101

ELECTRICITY IN THE DIAGNOSIS OF DISEASES OF THE NERVOUS SYSTEM. (PETERSON.)

Fig. 1.—Wire Brush Electrode . . . . . . . . . . . . . . . . . 149

Fig. 2.—Flat Sponge Electrode . . . . . . . . . . . . . . . . . 151

Fig. 3.—Interrupting Handle Electrode . . . . . . . . . . . . 151

Fig. 4.—Interrupting Handle Electrode . . . . . . . . . . . . . 152

Fig. 5.—Faradic Battery . . . . . . . . . . . . . . . . . . . . 154

Fig. 6.—Faradic Battery . . . . . . . . . . . . . . . . . . . . 154

Fig. 7.—Galvanic Battery . . . . . . . . . . . . . . . . . . . . 155

PATHOLOGY AND TREATMENT OF CLUB-FOOT. (JUDSON.)

Figs. 1 to 4 (inclusive.)—Retentive Lever Splints for Club-Foot . 156

Figs. 5 to 11 (inclusive )—Braces and Straps for Club-Foot . . . . 158

Fig. 12.—Piece of Webbing with Pad for Club-Foot . . . . . . . 161

Figs. 13 to 20 (inclusive.)—Braces and Straps for Club-Foot . . 162-163

IRRIGATION OF THE URETHRA AND BLADDER BY POSTURE AND CONTINUOUS CURRENT. (DAGGETT.)

Fig. 1.—Kiefer Nozzle . . . . . . . . . . . . . . . . . . . . . 455

Fig. 2.—Daggett Cannula . . . . . . . . . . . . . . . . . . . . 455

Fig. 3.—Daggett Examining Table . . . . . . . . . . . . . . . . 457

BUFFALO UNIVERSITY, MEDICAL DEPARTMENT, facing page . . . . . . . 517

ADENOMA OF THE NASO-PHARYNX. (CLARK.) . . . . . . . . . . . . . .

Gottstein Ring-Messer. Modified by Lefferts . . . . . . . . . . . . 595

NIAGARA UNIVERSITY, MEDICAL DEPARTMENT, facing page . . . . . . . 645

Shield to Protect the Clinical Thermometer. (WILLIAMS.) . . . . . . . 683

### Society Meetings.

The American Social Science Association will hold its next general meeting at Saratoga, beginning Monday, August 29th, and closing Friday, September 2, 1892. On Wednesday, August 31st, the Department of Health will hold forth with the following programme:

9.00 A. M., remarks by the Chairman of the Department, Frederick Peterson, M. D., of New York; 9.30 A. M., a report by the Secretary of the Department, W. D. Granger, M. D., on the Work of the Health Department since its Organization; 10.00 A. M., a paper by Matthew D. Field, M. D., of New York, on the Examination and Commitment of the Public Insane in New York City; 10.30 A. M., a paper by Ralph L. Parsons, M. D., of Sing Sing, N. Y., on Voluntary Commitment of the Insane to Asylums; 11.00 A. M., discussion of the preceding papers; 12.00 M., a paper by Henry Ling Taylor, M. D., of New York, on American Children, Hygienically Considered, followed by debate; 8.00 P. M., an address by W. W. Keen, M. D., of Philadelphia, on the Modern Surgery of the Brain; 9.00 P. M., a paper by Frederick Peterson, M. D., of New York, on the Old and the New Phrenology.

---

The American Microscopical Society will hold its fifteenth annual meeting at Rochester, N. Y., Tuesday, Wednesday, and Thursday, August 9, 10, 11, 1892.

---

The Mississippi Valley Medical Association will hold its eighteenth annual session at Cincinnati, Wednesday, Thursday, and Friday, October 12, 13, and 14, 1892, under the presidency of Charles A. L. Reed, M. D., Cincinnati. An excellent programme, containing the best names in the valley and covering the entire field of medicine, will be presented. An address on Surgery will be delivered by Dr. Hunter McGuire, of Richmond, Va., President of the American Medical Association. An address on Medicine will be made by Dr. Hobart Amory Hare, Professor of Therapeutics and Clinical Medicine, Jefferson Medical College, Philadelphia. The social as well as the scientific part of the meeting will be of the highest order. The officers of the Pan-American Medical Congress will hold a conference at the same time and place.

THE Medical Society of Wyoming County met at Portage, on Thursday, July 14, 1892, for scientific work. Dr. Robert Rae, of Portageville, was elected President; Dr. George S. Staif, of Gainsville, Vice-President; and Dr. P. S. Goodwin, of Perry Center, Secretary and Treasurer. The next regular meeting will be held at Silver Lake, September 8th, when a large attendance is expected.

THE American Public Health Association will hold its twentieth annual meeting in the City of Mexico, Tuesday, Wednesday, Thursday, and Friday, November 29th and 30th, and December 1 and 2, 1892. A large attendance is expected and special excursion rates are arranging.

Prominent sanitarians and scientists from the United States, Canada, Mexico, Central and South American Republics, will take active part in the meeting. The President is Dr. Felix Formento, of New Orleans, and the Secretary is Dr. Irving A. Watson, of Concord, N. H., who will furnish any further information desired.

Dr. Edward Clark, of Buffalo, is a member of the Executive Committee, who can also be consulted on any subjects relating to the meeting. Dr. F. P. Vandenburg, of Buffalo, is a member of the Committee on Polution of Water Supplies. Dr. Edward Clark, of Buffalo, serves on the Committee of Disposal of Garbage and Refuse, and Dr. Walter D. Greene, of Buffalo, is a member of the Committee on the Cause and Prevention of Diphtheria. It will be observed that our city is prominently identified with the conduct of affairs in this prominent medical organization.

NOTICE TO CONTRIBUTORS.—We are glad to receive contributions from every one who knows anything of interest to the profession. Articles designed for publication in the JOURNAL should be handed in before the first day of the month. The Editors are not responsible for the views or opinions of contributors. All communications should be addressed to the Managing Editor, 284 FRANKLIN ST., BUFFALO, N. Y.

